# Comprehensive Application of Time-of-flight Secondary Ion Mass Spectrometry (TOF-SIMS) for Ionic Imaging and Bio-energetic Analysis of Club Drug-induced Cognitive Deficiency

**DOI:** 10.1038/srep18420

**Published:** 2015-12-17

**Authors:** Su-Chung Youn, Li-You Chen, Ruei-Jen Chiou, Te-Jen Lai, Wen-Chieh Liao, Fu-Der Mai, Hung-Ming Chang

**Affiliations:** 1 Institute of Medicine, College of Medicine, Chung Shan Medical University, Taichung 40201; 2 Department of Anatomy, Faculty of Medicine, Chung Shan Medical University, Taichung 40201; 3 Department of Anatomy and Cell Biology, School of Medicine, College of Medicine, Taipei Medical University, Taipei, 11031; 4 Department of Biochemistry and Molecular Cell Biology, School of Medicine, College of Medicine, Taipei Medical University, Taipei 11031, Taiwan

## Abstract

Excessive exposure to club drug (GHB) would cause cognitive dysfunction in which impaired hippocampal Ca^2+^-mediated neuroplasticity may correlate with this deficiency. However, the potential changes of *in vivo* Ca^2+^ together with molecular machinery engaged in GHB-induced cognitive dysfunction has never been reported. This study aims to determine these changes in bio-energetic level through ionic imaging, spectrometric, biochemical, morphological, as well as behavioral approaches. Adolescent rats subjected to GHB were processed for TOF-SIMS, immunohistochemistry, biochemical assay, together with Morris water maze to detect the ionic, molecular, neurochemical, and behavioral changes of GHB-induced cognitive dysfunction, respectively. Extent of oxidative stress and bio-energetics were assessed by levels of lipid peroxidation, Na^+^/K^+^ ATPase, cytochrome oxidase, and [^14^C]-2-deoxyglucose activity. Results indicated that in GHB intoxicated rats, decreased Ca^2+^ imaging and reduced NMDAR1, nNOS, and p-CREB reactivities were detected in hippocampus. Depressed Ca^2+^-mediated signaling corresponded well with intense oxidative stress, diminished Na^+^/K^+^ ATPase, reduced COX, and decreased 2-DG activity, which all contributes to the development of cognitive deficiency. As impaired Ca^2+^-mediated signaling and oxidative stress significantly contribute to GHB-induced cognitive dysfunction, delivering agent(s) that improves hippocampal bio-energetics may thus serve as a promising strategy to counteract the club drug-induced cognitive dysfunction emerging in our society nowadays.

Gamma-hydroxybutyric acid (GHB) is a new club drug with highly addictive potential among adolescents^1^,^2^. Due to its significant pleasurable property, excessive consumption of GHB would cause numerous negative effects on neurological, cardiovascular and metabolic functions^3^,^4^,^5^. A variety of studies have indicated that unwarranted exposure to GHB (or its prodrug) would lead to cognitive dysfunction in which spatial memory impairment, decreased operant learning, and reduced psychomotor performance were well reported in rats, primates, and humans, respectively^6^,^7^,^8^,^9^. However, despite the abuse and prevalence of GHB is an on-going public health problem in our society, the mechanism(s) of GHB-induced cognitive dysfunction is still not well documented^10^. Within the past few years, the impaired Ca^2+^-mediated neuroplasticity in the hippocampus has been suggested to be positively correlated with the formation of cognitive deficiency^11^,^12^,^13^. Through extensively interrupting the Ca^2+^-mediated signaling, hippocampal neurons would suffer from bio-energetic dysfunction, which subsequently leads to the disruption of synaptic plasticity^11^. Seeing that criminal events (e.g. sexual assault) or traumatic accidents induced by GHB-relevant cognitive dysfunction has increasingly become an emerging problem^14^,^15^, exploring the molecular mechanisms underlying GHB-induced cognitive dysfunction would not only help us to better understand the neuro-toxic effects of GHB but also provide important insights to prevent or decrease the social costs arisen from GHB abuse nowadays^16^.

Long-term potentiation (LTP) is a molecular phenomenon characterized by pronounced synaptic transmission that plays an important role in memory formation and cognitive activity^17^,^18^,^19^. The *N*-methyl-D-aspartate (NMDA) receptor-mediated Ca^2+^ influx into post-synaptic neurons is the critical event leading to the induction of LTP^20^,^21^. Increased Ca^2+^ level in post-synaptic neurons would trigger nitric oxide production, which regulates downstream gene expression through cAMP-responsive element binding protein (CREB) phosphorylation as well as positively backs to the pre-synaptic terminal and enhances glutamate release^22^,^23^. This long-lasting enhancement of synaptic transmission strengthens the hippocampal neuroplasticity, which forms the cellular basis for memory consolidation and cognitive activity^17^,^18^,^19^. Previous study has indicated that repeated GHB exposure would depress the NMDA receptor activity in cerebral cortex^24^. Pharmacological reports also demonstrated that excessive high levels of GHB would cause oxidative stress that consequently results to learning and memory dysfunction^25^,^26^. As both LTP and oxidative status play an important role in modulating hippocampal function, understanding the bio-energetic regulation as well as the spatio-temporal integration of related elements engaged in neuroplasticity would therefore be a promising strategy for clinical design of therapeutic agent(s) to counteract the GHB-related cognitive deficiency.

However, although the functional role of Ca^2+^-mediated signaling in potentiating the hippocampal neuroplasticity has been well documented, the potential changes of *in vivo* Ca^2+^, together with the molecular machinery involved in neuroplastic regulation following GHB has never been reported. Moreover, whether GHB-induced cognitive dysfunction is effectively attributed to bio-energetic impairment subsequently to enhanced oxidative stress still remains to be further explored. Considering that the non-penetrative and probe-free time-of-flight secondary ion mass spectrometry (TOF-SIMS) is a powerful surface analysis technique capable of providing the fingerprintable mass spectral information as well as imaging ionic expression with high sensitivity and excellent spatial distribution in biological samples^27^,^28^,^29^,^30^, the present study is firstly aimed to determine the potential neuroplastic changes induced by GHB through molecular imaging, spectrometric, biochemical, neurochemical, as well as behavioral approaches. Secondly, as oxidative stress disrupts neuronal function, and contributes to cognitive deficiency^31^,^32^, the extent of oxidative injury, along with the changes of cognitive expression were further assessed by malondialdehyde level (MDA), and Morris water maze test, correspondingly. Finally, as an attempt to correlate all the molecular and biochemical alterations induced by GHB with the functional impairment of hippocampal bio-energetics, the Na^+^/K^+^ ATPase, cytochrome oxidase (COX), and [^14^C]-2-deoxyglucose (2-DG) activities were further processed in the current study.

## Results

**Figure 1:**
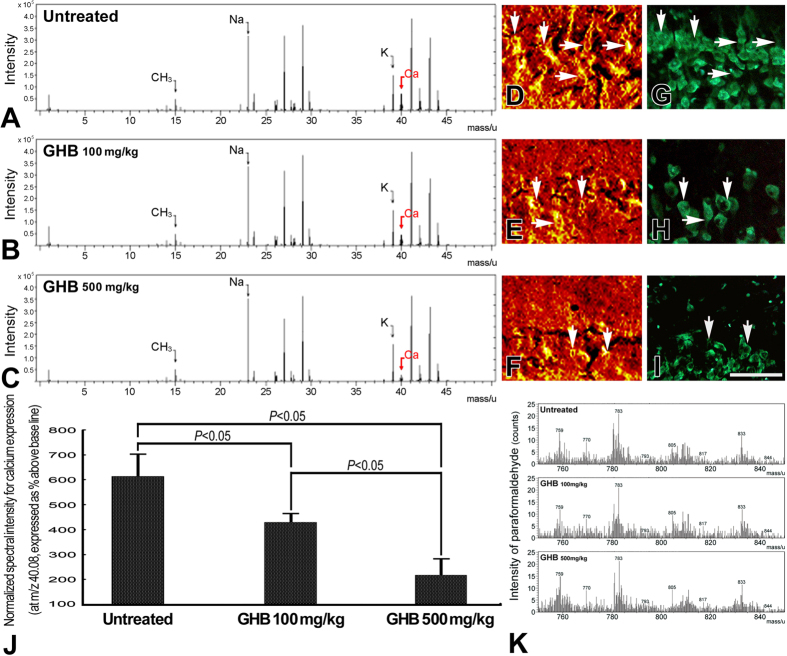
TOF-SIMS positive spectra (**A–C**), ionic imaging (**D–F**), calcium green 1 fluorescence photomicrographs (**G–I**), and histogram (**J**) showing the hippocampal Ca^2+^ expression in the normal untreated (**A,D,G**), γ-hydroxybutyric acid (GHB) 100 mg/kg intoxicated (**B,E,H**), and GHB 500 mg/kg intoxicated rats (**C,F,I**). The ionic imaging of Ca^2+^ signaling is detected at the coronal plane located 4.36 mm posterior to the bregma (i.e. 4.64 mm anterior to the interaural line), and expressed by a color scale in which bright colors represent high levels of Ca^2+^. Note that in normal untreated rats, strong calcium intensity with significant intracellular localization was detected in hippocampal neurons (arrows) (**D,G**). However, following different doses of GHB intoxication, the hippocampal Ca^2+^ expression was drastically decreased in both terms of ionic/histological imaging (**E,F,H,I**) and spectral intensity (**B,C**). The maximal reduction of Ca^2+^ expression was observed in animals receiving higher dose of GHB (500 mg/kg) (**C,F,I**). Data from normalized spectral intensity analysis corresponded well with imaging findings in which GHB intoxication effectively decreased the hippocampal Ca^2+^ intensity (**J**). Also note that the stability of the paraformaldehyde (PFA) signal among different experimental groups was clearly demonstrated by yielding similar spectral profiles around the *m/z* of 760 ~ 840 (**K**). Scale bar = 100 μm in both ionic and histological imaging.

**Figure 2:**
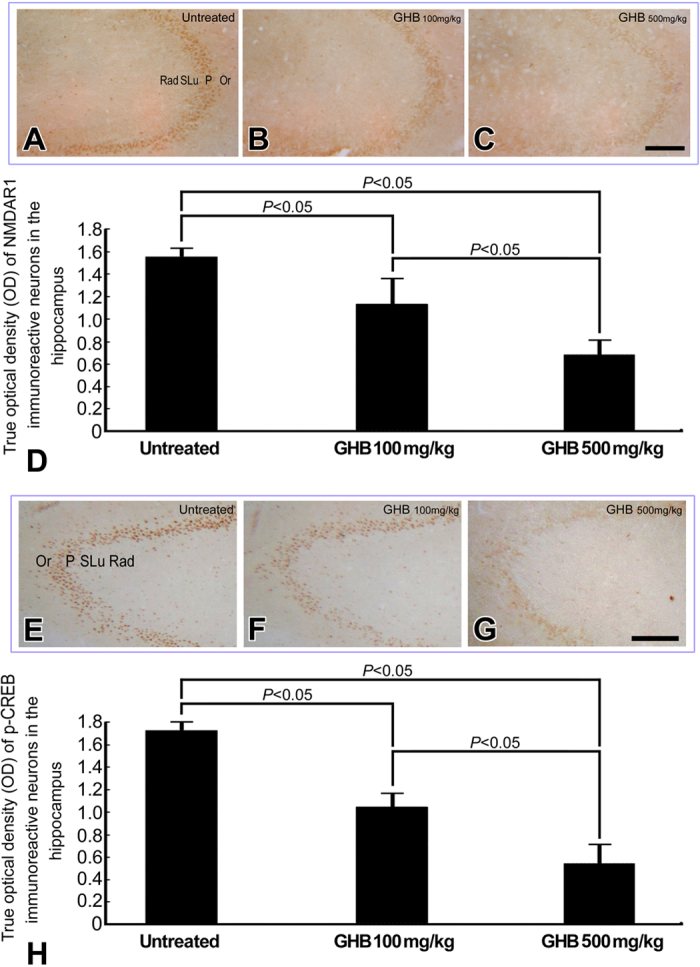
Light photomicrographs (**A–C,E–G**) and histograms (**D,H**) showing the hippocampal *N*-methy-D-aspartate receptor subunit 1 (NMDAR1) (**A–D**) and the phosphorylated cAMP- responsive element binding protein (p-CREB) (**E-H**) immunoreactivities in the normal untreated (**A,E**), γ-hydroxybutyric acid (GHB) 100 mg/kg intoxicated (**B,F**), and GHB 500 mg/kg intoxicated rats (**C,G**). Note that in normal untreated rats, numerous NMDAR1 and p-CREB immunoreactive neurons with strong staining intensities were observed in hippocampal pyramidal cell layers (**A,E**). However, following different doses of GHB intoxication, the immuno-expression of both hippocampal NMDAR1 and p-CREB were drastically decreased in which the lowest expression was detected in animals received 500 mg/kg of GHB (**C,G**). Computerized image analysis corresponded well with immunohistochemical findings in which the optical density of NMDAR1 and p-CREB immunoreactive neurons was significantly reduced in GHB-treated animals (**D,H**). Rad: stratum radiatum; SLu: stratum lucidum; P: pyramidal cell layer; Or: stratum oriens. Scale bar = 200 μm. (**A–C,E–G**).

**Figure 3:**
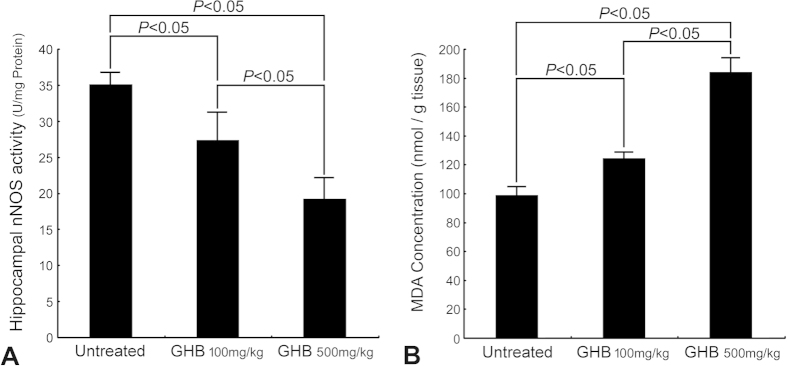
Histograms showing the neuronal nitric oxide synthase (nNOS) activity (**A**) and the extent of oxidative stress [as demonstrated by malondialdehyde (MDA) level] (**B**) in the hippocampus of normal untreated, γ-hydroxybutyric acid (GHB) 100 mg/kg intoxicated, and GHB 500 mg/kg intoxicated rats. Note that in normal untreated rats, the nNOS activity was measured to be 35 ± 1.74 U/mg protein (**A**). However, in animals subjected to different doses of GHB, the hippocampal nNOS activity was drastically decreased (**A**). Also note that only relative low level of oxidative stress was detected in the hippocampus of normal untreated rats (**B**). However, following different doses of GHB intoxication, the extent of hippocampal oxidative stress was severely increased (**B**). The alterations of these biochemical parameters induced by GHB were dose-dependent since the maximal change was observed in animals given higher dose of GHB (500 mg/kg).

**Figure 4:**
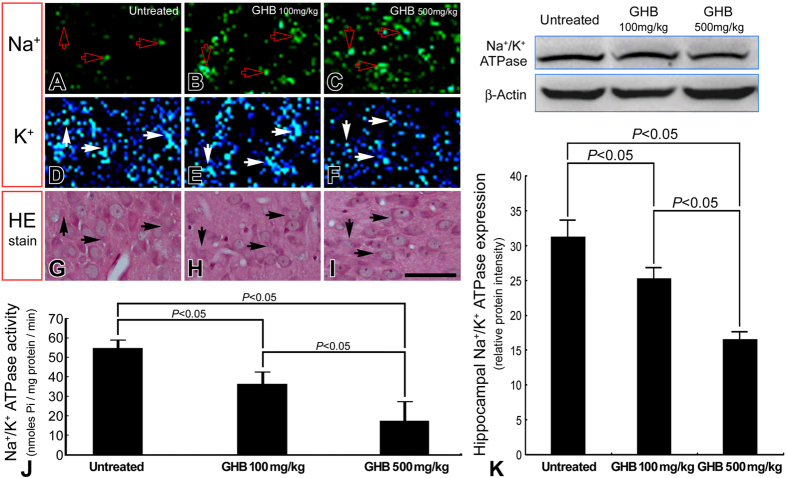
TOF-SIMS ionic imaging (**A–F**), photomicrographs of hemotoxylin-eosin (H&E) staining (**G–I**), immunoblots (**K**), and histograms (**J,K**) showing the intracellular Na^+^ (**A–C**), K^+^ (**D–F**), Na^+^/K^+^ ATPase activity (**J**) as well as its expression (**K**) in the hippocampus of normal untreated (**A,D,G**), γ-hydroxybutyric acid (GHB) 100 mg/kg intoxicated (**B,E,H**), and GHB 500 mg/kg intoxicated rats (**C,F,I**). The ionic/histological imaging is detected at the granular layer of dentate gyrus from coronal plane located 4.36 mm posterior to the bregma and 4.64 mm anterior to the interaural line. Note that in normal untreated rats, mild Na^+^ (**A**) and intense K^+^ (**D**) signaling were detected in the intracellular portion (arrows in (**A,D,G**) of hippocampal neurons. The relative low level of intracellular Na^+^ corresponded well with the normal activation (**J**) and higher amounts of Na^+^/K^+^ ATPase activity (**K**). However, following different doses of GHB intoxication, both the activity (**J**) and the amount (**K**) of Na^+^/K^+^ ATPase was drastically decreased. The impairment of Na^+^/K^+^ ATPase (**J,K**) was visibly expressed by disrupted ionic regulation in which excessive accumulation of intracellular Na^+^ (arrows in (**B,C,H,I**) in association with deficient intracellular K^+^ (arrows in E,F,H,I) was clearly observed in hippocampal neurons. Scale bar = 100 μm. (**A–I**).

**Figure 5:**
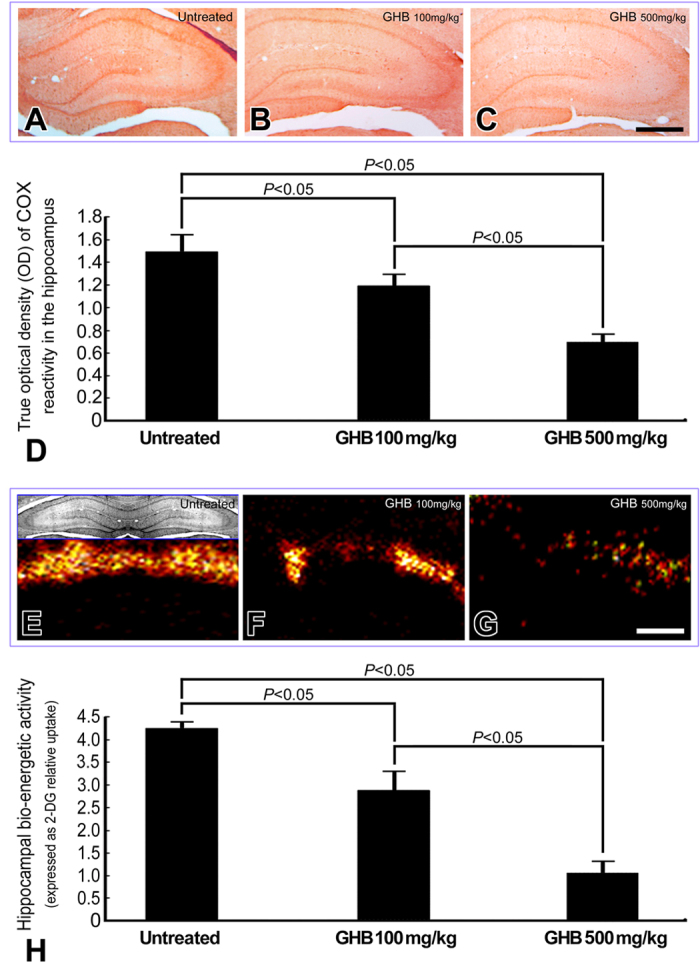
Light photomicrographs (**A–C**), colored autoradiograms (**E–G**), and histograms (**D,H**) showing the bio-energetic status [as demonstrated by cytochrome oxidase (COX) reactivity (**A–D**) and [^14^C]-2-deoxyglucose (2-DG) activity (**E-H**), respectively] in the hippocampus of normal untreated (**A,E**), γ-hydroxybutyric acid (GHB) 100 mg/kg intoxicated (**B,F**), and GHB 500 mg/kg intoxicated rats (**C,G**). Note that in normal untreated rats, numerous neurons with strong COX staining (**A**) and intense radioactive signals for 2-DG (**E**) was expressed in the hippocampal regions. However, following different doses of GHB intoxication, the hippocampal COX (**B,C**) and the signal intensity of 2-DG (**F,G**) was drastically decreased. Quantitative evaluation showed that the depressive effect of GHB on hippocampal bio-energetics was dose-dependent since the maximal reduction was detected in animals exposed to GHB at 500 mg/kg (**D,H**). Scale bar = 600 μm in (**A–C**); = 1200 μm in (**E–G**).

**Figure 6:**
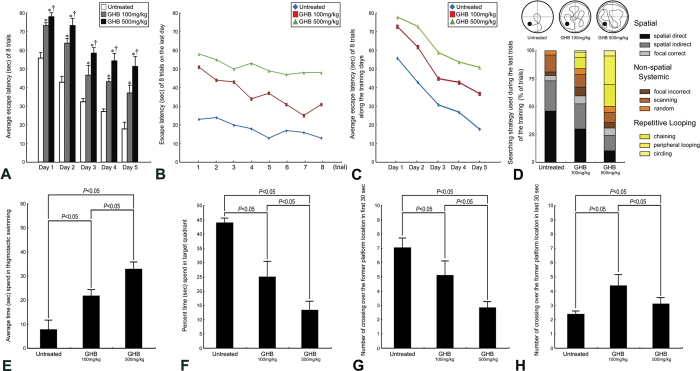
Histograms (**A,D,E,F,G,H**) and line charts (**B,C**) showing the cognitive function [as determined by the Morris water maze test] of normal untreated, γ-hydroxybutyric acid (GHB) 100 mg/kg intoxicated, and GHB 500 mg/kg intoxicated rats. Note that the average escape latency to find the hidden platform over five consecutive days was significantly higher in the GHB-intoxicated rats (**A**). When compared to normal untreated rats, rats intoxicated by GHB also showed a slower learning curve either over the 8 trials on the last training day (**B**) or along all the training days (**C**). Inspection of the swimming paths during the last trials of training further displayed that GHB impaired task acquisition by increasing the probability to use non spatial searching strategies (**D**). Thigmotactic behavior was also evident in GHB-intoxicated rats (**E**). Also note that in the probe test, there was a significant impairment of cognitive function following different levels of GHB intoxication as revealed by reducing the percent time spent in the target quadrant (**F**) and decreasing the number of crossings over the former platform location (G). The cognitive flexibility of GHB-intoxicated rats was also impaired in which these rats rarely shift their searching strategy to find the former platform during the last seconds of probe test. (**H**). *P < 0.05 as compared to normal untreated values; ^†^*P* < 0.05 as compared to GHB 100 mg/kg values (**A**).

In normal untreated rats, strong Ca^2+^ intensities with significant intracellular localization were detected in hippocampal neurons by the use of ionic imaging, calcium green-1, and TOF-SIMS analysis (Fig. 1A,D,G). The enhanced Ca^2+^ expression in hippocampal neurons indicated that the intracellular Ca^2+^-mediated signaling was intact to maintain normal neuroplasticity and neuronal functions. Data collected from quantitative immunohistochemical and biochemical studies corresponded well with these findings in which an intense NMDA receptor expression (Fig. 2A,D), high level of nNOS activity (Fig. 3A), and enhanced CREB phosphorylation (p-CREB) (Fig. 2E,H) were all clearly detected in hippocampus of normal untreated animals. In addition, the well-expressed Ca^2+^-mediated NMDA receptor activation and nNOS-activated CREB phosphorylation was positively correlated with the intact bio-energetics in which lower level of oxidative stress (Fig. 3B), normal activation and large amounts of Na^+^/K^+^ ATPase (Fig. 4A,D,J,K), pronounced expression of COX (Fig. 5A,D), and strong radioactive signals of 2-DG (all are reliable markers of bio-energetics) (Fig. 5E,H) were evidently demonstrated in hippocampal neurons of normal untreated rats. Behavioral data coincided well with above findings in which animals expressed higher bio-energetics exhibited good performances in Morris water maze test of spatial learning [i.e. with short average escape latency (Fig. 6A), quick learning curve (Fig. 6B,C), and frequently use of spatial searching strategy (Fig. 6D) during the training session] and spatial memory [i.e. spent large time in target quadrant (Fig. 6F) and increased the number of crossings over the former platform location in first seconds of probe test (Fig. 6G)]. It is noteworthy that in normal untreated rats, just a few seconds of thigmotactic swimming was detected (Fig. 6E). As regard to the expression of cognitive flexibility, the performance of normal untreated rats was also good as these rats showed a rapid shift of the searching strategy in last periods of the probe test (Fig. 6H).

However, in animals subjected to chronic and excessive doses of GHB, the hippocampal Ca^2+^ expression was drastically decreased in both terms of spectral intensity and ionic/histological imaging (Fig. 1B,C,E,F,H,I). The decrement of hippocampal Ca^2+^ was suggested to be correlated with depressed Ca^2+^-mediated signaling, which was supported by quantitative findings wherein a diminished NMDA receptor expression (Fig. 2B–D), lower level of nNOS activity (Fig. 3A), and reduced p-CREB immunoreactivity (Fig. 2F–H) were all detected in hippocampus of animals intoxicated by GHB. Biochemical and auto-radiographic measurements corresponded well with these molecular findings in which severe oxidative stress (Fig. 3B), decreased Na^+^/K^+^ ATPase protein level and activity (Fig. 4J,K), impaired COX expression (Fig. 5B–D), and reduced 2-DG signaling (Fig. 5F–H) were all clearly observed in the hippocampus of animals subjected to GHB. The impairment of Na^+^/K^+^ ATPase was clearly expressed by ionic imaging (Fig. 4A–F) wherein unexpected high levels of Na^+^ were accumulated in intracellular portion of hippocampal neurons instead of pumping outward (arrows in Fig. 4B,C,H,I, with using H&E stain as a morphological reference). Data collected from behavioral testing paralleled to these findings in which animals bearing with impaired hippocampal bio-energetics (Figs 3B,4 and 5) definitely showed poor performances in cognitive activity (Fig. 6). Spatial learning tests indicated that long average escape latency (Fig. 6A), slow learning curve (Fig. 6B,C), and mainly employed of non-spatial searching strategies (Fig. 6D) were detected in animals intoxicated by different doses of GHB. The GHB-intoxicated rats also showed anxiogenic behavior as these rats spent much time in thigmotactic swimming rather than found the hidden platform straightforwardly (Fig. 6E). The establishment of spatial memory and cognitive flexibility were also impaired following GHB in which rats received GHB spent less time in target quadrant (Fig. 6F) and rarely shift their searching strategy in last seconds of the probe test (Fig. 6H). Nevertheless, it is noteworthy that the cognitive deficits observed in behavioral testing did not result from potential motoric dysfunction induced by GHB, since GHB (at least in our dosages) did not affect the cued task performance in Morris water maze testing [i.e. no significant difference was detected in swimming speed between normal untreated rats (27.56 ± 1.43 cm/min.) and GHB-treated ones (25.19 ± 2.11 cm/min.)].

## Discussion

The present study provides the first functional anatomical evidence that chronic and excessive GHB exposure would decrease hippocampal Ca^2+^ expression (Fig. 1), depress Ca^2+^-mediated molecular element activation (Figs 2 and 3A), enhance oxidative stress (Fig. 3B), impair bio-energetics (Figs 4 and 5), and ultimately contribute to the development of cognitive deficiency (Fig. 6). The detrimental effects of GHB was dose dependent, since the most apparent changes were detected in animals subjected to higher dose of GHB (Figs 1, 2, 3, 4, 5, 6). It was known that Ca^2+^ is an essential element participated in the induction of LTP^20^. Through ion channels mediated by post-synaptic NMDA receptors, extensive influx of Ca^2+^ would trigger nNOS activation, which induces downstream CREB phosphorylation and produces NO that retrogrades to pre-synaptic terminals and effectively strengthen the synaptic plasticity^21^,^22^,^23^. Pathological studies have indicated that dyshomeostasis of Ca^2+^ in hippocampus would depress the efficacy of LTP that consequently leads to the impairment of cognitive function^33^,^34^. Our previous study also demonstrated that decreased hippocampal Ca^2+^ could serve as the ionic mechanism underlying the formation of sleep deprivation-relevant cognitive deficiency^12^. This is justly the case that in our current study, we also detected a reduced Ca^2+^ expression in the hippocampus following GHB (Fig. 1). The reduction of Ca^2+^ is positively correlated with the poor performances in spatial learning and spatial memory (Fig. 6). Although the detailed mechanism of GHB-induced cognitive dysfunction is not fully understood, our present study clearly demonstrated that depressed hippocampal Ca^2+^-mediated signaling (Figs 2 and 3A) secondary to reduced hippocampal Ca^2+^ (Fig. 1) would play a much important role in the pathogenesis of GHB-induced cognitive deficiency (Fig. 6). To the best of our knowledge, there is no report designed to systemically examine the potential impacts of GHB on molecular machinery involved in the induction of LTP. Moreover, due to limitation of conventional methodologies, the *in vivo* Ca^2+^ expression, together with the Ca^2+^-mediated signaling following GHB has never been explored previously. As the present study is the first one employing advanced mass spectrometry and biochemical approaches to clearly display the reduced changes of hippocampal Ca^2+^ as well as the depressive Ca^2+^-mediated signaling after GHB, delivering agent(s) that can preserve the hippocampal Ca^2+^ and/or modulate the Ca^2+^-mediated signaling may therefore serve as a practical strategy to counteract the GHB-induced cognitive sequelae.

In addition to depress the molecular signaling in hippocampal neurons, the harmful effects of GHB on producing oxidative stress and interrupting the cellular bio-energetics should not be overlooked. Enhanced oxidative stress could cause lipid peroxidation that inevitably disrupts the function of hippocampal Na^+^/K^+^ ATPase, an important membrane-bound enzyme responsible for the maintenance of ionic gradient, cell volume and neuronal excitability^31^,^32^,^35^,^36^. Reduction of hippocampal Na^+^/K^+^ ATPase has been reported to impair downstream molecular signaling, and exert deleterious consequences on the mechanisms of learning and memory^37^,^38^. The present study coincided well with previous findings in which we also detected a decreased Na^+^/K^+^ ATPase expression (in both terms of protein level and enzymatic activity, Fig. 4J,K), depressive Ca^2+^-mediated signaling (Figs 2 and 3A), and significant cognitive dysfunction (as revealed by use of non spatial searching strategies and less behavioral flexibility in the water maze, Fig. 6) in those animals intoxicated by GHB. The reduction of Na^+^/K^+^ ATPase expression was clearly demonstrated by impaired ionic transporting across cell membranes in which excessive Na^+^ were accumulated in intracellular portion of hippocampal neurons instead of pumping outward (arrows, Fig. 4B,C,H,I, with using H&E stain as a morphological reference). In addition to depress the molecular signaling in neuroplastic regulation, intracellular Na^+^ overload would also suppress the terminal enzyme of mitochondrial electron transport chain (i.e. COX) (Fig. 5B–D), which consequently disrupts neuronal bio-energetics (Fig. 5F–H) and effectively contributes to the development of cognitive deficiency (Fig. 6). As the molecular link between GHB-induced cognitive dysfunction and impaired hippocampal bio-energetics secondary to enhanced oxidative stress has been clearly demonstrated in this study, application of anti-oxidative agent may therefore shed an important light for clinical use to improve or rescue the cognitive function in GHB-intoxicated victims.

Another important issue to be addressed is the dose of GHB administrated in the current study. GHB might exert distinct effects depending on its concentrations^39^. Injection of lower dose of GHB (e.g. endogenous physiological concentration) causes short-term antegrade amnesia or minor neuro-toxic effects^39^,^40^. In contrast, considerable amounts of neuronal impairments and cognitive dysfunction could be detected in animals receiving very low, supra-physiological or supra-pharmacological levels of GHB^5^,^10^,^24^,^25^. Although the detailed mechanisms involved in producing the biphasic effect of GHB have not been well-documented, alterations in neuronal excitability mediated by NMDA receptor and Na^+^/K^+^ ATPase may partially account for the occurrence of this discrepancy^41^,^42^. It has been reported that repeated and high dose of GHB could depress the excitability of cortical glutamatergic system, an effect not detected in animals subjected to physiological dose of GHB^39^,^42^. Similar findings were also detected in our present study in which remarkable cognitive deficits (Fig. 6) induced by supra-pharmacological level of GHB was strongly correlated with the reduction of hippocampal NMDA receptor expression (Fig. 2B–D), Na^+^/K^+^ ATPase dysfunction (Fig. 4J,K), and bio-energetic destruction (Fig. 5), all are reliable markers for general depression of neuronal excitability. Nevertheless, caution must be exercised when discussing the cognitive impacts of GHB since GHB has been reported to exert either anxiolytic or anxiogenic effects on behavioral activity^43^,^44^. It is well documented that the anxiety state of the subjects is one of the major factors accounting for part of the variability in learning and memory abilities^45^,^46^. Animals that consistently express high levels of anxiety usually display poor performances in learning and memory^47^,^48^. As the memory storage and the anxiety processes may interact by using some common neural substrates or by modulating each other in an adaptative way^45^, any deficit observed in the cognitive task may partially be related to the anxious nature of GHB, but not only directly to the negative effects of GHB on cognitive activity. This seems to be the case that in our current study, we also observed a significant correlation between the time rats spent in thigmotactic swimming (i.e. anxious behavior) and the cognitive impairment in both the acquisition and retrieval of spatial message (Fig. 6). Although we did not know exactly to what extent does the anxiogenic action of GHB affect the cognitive performance in our current experimental paradigm, the present study thus provides new avenues for considering the neurobiological mechanisms linking anxiety and memory processing in the investigation of GHB-induced cognitive deficiency. On the other hand, it is noteworthy that the neuro-protective effects of GHB under pharmacological levels have also been reported^41^,^49^. Although the underlying mechanisms for this discrepancy still remains unclear, the treatment period of GHB (single dose vs. multiple injections), age picked for receiving GHB (adolescent vs. adult), animal species used for GHB (rabbit vs. rat), and even the lesion model may all account for the variance observed between our results and those reported previously.

**Figure 7:**
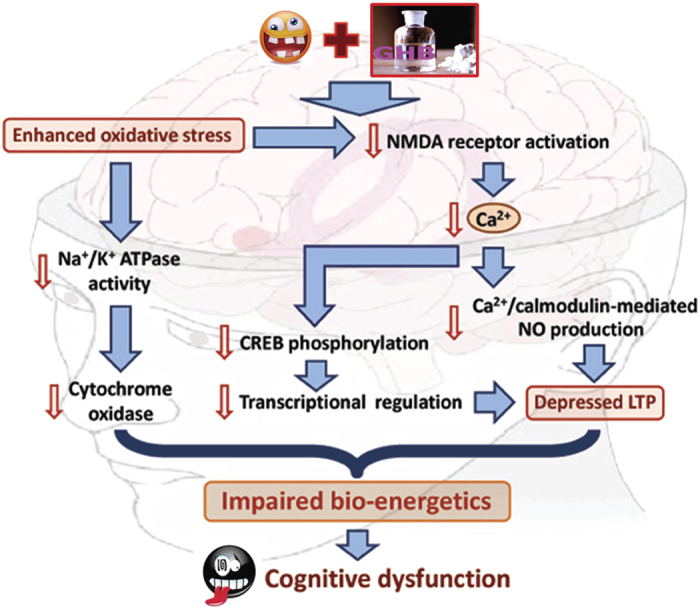
Schematic diagram showing the possible mechanism(s) of GHB-induced cognitive dysfunction. Note that excessive exposure to GHB would cause oxidative stress and depress long-term potentiation (LTP), which synergistically impair hippocampal bio-energetics and contribute to the formation or development of cognitive deficiency. Background image: modified from https://commons.wikimedia.org/wiki/File:Hippolobes.gif. Smiley emoji: modified from https://commons.wikimedia.org/wiki/File:Ras.gif, and https://commons.wikimedia.org/wiki/File:Tired_Face.png.

In summary, with the assistance of advanced spectrometric, ionic imaging, biochemical, morphological as well as behavioral approaches, the present study addressed for the first time that chronic and excessive exposure to GHB would cause cognitive dysfunction in which impaired hippocampal bio-energetics may contribute to the pathogenesis of this deficiency (Fig. 7). Although the detailed mechanism(s) related to bio-energetic dysfunction is still not fully understood, depressed Ca^2+^-mediated signaling and reduced Na^+^/K^+^ ATPase expression resulted from enhanced oxidative stress may underlie the formation of such sequelae. Considering that maintaining hippocampal bio-energetics is necessary for normal cognitive regulation, delivering agent(s) that could preserve Ca^2+^-mediated signaling and/or modulate oxidative/ionic homeostasis may thus serve as a novel strategy for clinical treatment of cognitive dysfunction induced by increasingly prevalence of GHB abuse nowadays.

## Materials and Methods

### Treatment of experimental animals

Adolescent male Wistar rats (n = 36, postnatal day 42) obtained from the Laboratory Animal Center of the Chung Shan Medical University were used in this study. The experimental animals were divided into three groups equally. Rats in the first group were intraperitoneally injected with Ringer’s solution for ten successive days. Rats in the second and third group were daily injected with GHB (for ten successive days) at the doses of 100 and 500 mg/kg, respectively. GHB (courtesy of the Food and Drug Administration, Ministry of Health and Welfare, Taipei, Taiwan) was prepared in physiological saline for each batch of rats. During the experimental period, all rats were exposed to an automatically regulated light-dark cycle of 12:12 at a constant temperature of 25 ± 1 °C. The animals were allowed to food and water *ad libitum*. In the care and handling of all experimental animals, the Guide for the Care and Use of Laboratory Animals (1985) as stated in the United States NIH guidelines (NIH publication No. 86-23) were followed. All the drug administration procedures were further approved by the Committee on Care and Use of Laboratory Animals of the Chung Shan Medical University (IACUC Approval No 9420).

### Morris water maze learning test

Started from the fifth day of Ringer’s or GHB consecutive administration, all rats were undergone the Morris water maze test for examining the performance of spatial learning and memory^50^,^51^. The Morris water maze was consisted of a circular tank (80 cm deep, 164 cm diameter, San Diego Instruments, CA, USA) filled water to a depth of 24 cm. The pool water was made opaque by addition of semi-skimmed milk. The maze was divided into four equal quadrants on the monitoring screen of a computer. An escape platform placed in one of the quadrants (target quadrant) was submerged below the water surface. The rats were trained to find the hidden platform according to the spatial cues in the experimental room. Place learning was assessed for eight training trials per day (with the inter-trial interval of 30 s) for five consecutive days 30 min after GHB exposure. The searching strategy was analyzed during the last trials of the training session. An independent investigator blinded to the experimental groups assigned a predominant searching strategy for each trial using the categorization developed previously: spatial strategies, involving spatial direct, spatial indirect and focal correct strategies; non-spatial systemic strategies, involving scanning, random and focal incorrect strategies; and strategies involving repetitive looping, i.e. chaining, peripheral looping and circling strategies^52^,^53^. Thigmotactic swimming was quantified by dividing the maze into two circles and the times spent in the outer circle of the pool (22 cm wide) was designated as thigmotactic behavior. The maze performance was recorded by a video camera suspended above the maze and interfaced with a video tracking system (San Diego Instruments, CA, USA).

A probe trial was given 12 h after training to test the extent of memory consolidation and cognitive flexibility^51^. The time spent in target quadrant and the number of crossings over the former platform location during the first 30 s of probe trial was used as measures to indicate the degree of memory consolidation that has taken place after learning. The number of crossings over the former platform location during the last seconds of probe trial was used to assess the extent of cognitive flexibility.

To test the possible deficits in motoric function after GHB, the rats were tested with a visible platform on a new location (the cued task). The swimming speed to reach the platform was also recorded for each trial.

### Perfusion and tissue preparation

For TOF-SIMS analysis, and quantitative morphological study, half amounts of rats from all experimental groups (n = 6 in each experimental group) were deeply anesthetized with 7% chloral hydrate and perfused transcardially with saline followed by 4% paraformaldehyde in 0.1 M phosphate buffer (PB). After perfusion, the hippocampus was removed and kept in sucrose buffer for cryoprotection at 4 °C overnight. Serial 30-μm-thick sections of the hippocampus were cut transversely with a cryostat (CM3050S, Leica Microsystems, Wetzlar, Germany) on the following day and were alternatively placed into four wells of a culture plate. Sections collected in the first well were processed for TOF-SIMS analysis, and those in second to fourth wells were processed for NMDA receptor subunit 1 (NMDAR1) immunohistochemistry, phosphorylated CREB (p-CREB) immunohistochemistry, and COX histochemistry, respectively.

### Preparation of hippocampal slices

For standard calcium staining, another half amounts of animals from all experimental groups were decapitated under deep anesthesia. The hippocampus was rapidly isolated and placed in ice-cold standard solution containing (in mM): 126 NaCl, 3.5 KCl, 2 CaCl_2_, 1.2 NaH_2_PO_4_, 1.3 MgCl_2_, 25 NaHCO_3_, 11 glucose, bubbled with 95% O_2_ and 5% CO_2_. Hippocampal slices of 200 μm thickness were cut using a vibratome and immediately transferred to an incubation chamber containing the same solution at 33 °C (pH 7.4). The intracellular Ca^2+^ was measured by immersing the hippocampal slices with the long wavelength calcium indicator calcium green-1 AM (Molecular Probes, Eugene, OR, USA) at the concentration of 10 μM for 45 min at 33 °C^54^. Calcium green-1 labelled neurons were visualized with a fluorescence microscope (ZEISS) equipped with a proper filter (450–490 nm for excitation). After photomicrography, the sections were rinsed in standard solution and then fixed with 4% paraformaldehyde for subsequent TOF-SIMS analysis.

### TOF-SIMS analysis

TOF-SIMS analysis was carried out on a TOF-SIMS IV instrument (ION-TOF GmbH, Münster, Germany) as described in our previous studies^12^,^55^. Fixed hippocampal slices or cryostated sections were attached to silica wafers and the temperature of sample holder was adjusted to −60°C. Gallium (Ga^+^) ion gun operated at 25 kV was used as the primary ion source (1 pA pulse current). The Ga^+^ primary ion beam was scanned over an area of 500 μm^2^ which including 128 × 128 pixels. Image data acquisition time was 200 s with the best resolution obtained at m/Δm = 7450. Positive secondary ions flying through a reflectron mass spectrometer were detected with a micro-channel plate assembly operating at 10 kV post-acceleration. The paraformaldehyde and a set of standard peaks [like *m/z* 15 (CH_3_^+^), 27 (C_2_H_3_^+^), and 41 (C_3_H_5_^+^)] were used as mass calibration to ameliorate the potential matrix effect for ion spectrums^55^. The ions related to *m/z* 23, *m/z* 39.09, and *m/z* 40.08 were used to identify and evaluate the ionic image of Na^+^, K^+^, and Ca^2+^, respectively.

### Quantitative NMDAR1 and p-CREB immunohistochemistry

For NMDAR1 and p-CREB immunohistochemistry, sections were first placed in 0.01 M phosphate buffer saline containing 10% methanol and 3% hydrogen peroxide for 1 h to reduce endogenous peroxidase activity. Following this, sections were incubated in blocking medium containing 0.1% Triton X-100, 3% normal goat serum and 2% bovine serum albumin (all from Sigma, St. Louis, MO, USA) for 1 h to block nonspecific binding. After that, sections were incubated with primary antibody against NMDAR1 (1:75) (Chemicon AB1516, Temecula, CA, USA) and p-CREB (1:100) (Cell Signaling 9198, Danvers, MA, USA) in blocking medium at 4 °C for 48 h. Lastly, sections were incubated with a biotinylated secondary antibody (1:200) (Vector Laboratories, Burlingame, CA, USA) at room temperature for 2 h, followed by standard avidin-biotin complex procedure with diaminobenzidine as a substrate of peroxidase.

### Cytochrome oxidase histochemistry

The reaction medium for COX histochemistry contained 0.03% cytochrome c, 0.05% 3,3’- diaminobenzidine and 0.02% catalase (all from Sigma, St. Louis, MO, USA) in 0.1 M PB, pH 7.4. The sections were incubated with this medium at 4 °C overnight. After incubation, sections were rinsed with distilled water to terminate the reaction.

### Measurement of hippocampal lipid peroxidation

Rats from all experimental groups were killed by deeply anesthesia and the hippocampus was excised immediately. After that, the hippocampus was homogenized and centrifuged for 10 min at 4 °C. The supernatants were then taken for detecting MDA by measurement of fluorescence product formed from the reaction of this aldehyde with thiobarbituric acid. The results were determined spectrophotometrically at 532 nm to represent MDA levels using tetraethoxypropane as a standard, and expressed as nmol/mg.

### Hippocampal nNOS activity assay

Total nNOS activity was determined by measuring the conversion of L-[^14^C]-arginine to L-[^14^C]-citrulline. Hippocampus taken from all experimental groups were homogenized and centrifuged for 15 min at 4 °C. The tissue extract (50 μL) was then incubated in reaction mixture containing 50 mM HEPES, 200 μM nicotinamide adenine dinucleotide phosphate (β-NADPH), 1 mM CaCl_2_, 50 μM tetrahydrobiopterin (BH4), and 1 μCi/ml of L-[^14^C]-arginine. The reaction was terminated by adding 1 mL of ice-cold 50 mM HEPES (pH 5.5). Subsequently, the tissue samples were applied to Dowex AG50W-X8 column to remove L-[^14^C]-arginine. The L-[^14^C]-citrulline was quantified by liquid scintillation spectrophotometry (Beckman LS 3801). 1 U of total nNOS activity was defined as picomoles of L-[^14^C]-citrulline produced per minute per microgram protein. Hippocampal nNOS activity was expressed as units per milligram of hippocampus protein (U/mg Protein).

### Hippocampal Na^+^/K^+^ ATPase activity assay

For hippocampal Na^+^/K^+^ ATPase activity assay, tissue samples were first homogenized in 10 volumes (1:10, w/v) of 0.32 mM sucrose solution containing 5 mM HEPES and 1 mM ethylenediaminetetraacetic acid (EDTA), pH 7.5. The homogenates were then centrifuged, and supernatants were used for Na^+^/K^+^ ATPase activity assay by reacting with 5 mM MgCl_2_, 80 mM NaCl, 20 mM KCl, and 40 mM Tris-HCl, pH 7.4, in a final volume of 200 μL. After pre-incubation at 37 °C, the reaction was initiated by addition of ATP to a final concentration of 3 mM, and incubated for 20 min. Controls were carried out under the same conditions with the addition of 1 mM of ouabain. Specific activity of Na^+^/K^+^ ATPase was expressed as nmol of inorganic phosphate (Pi) released per min per mg of protein.

### Immunoblotting analysis for hippocampal Na^+^/K^+^ ATPase level

For immunoblotting analysis, tissue samples were first homogenized with 100 μL lysis buffer using a grinder on ice. The immunoblot procedure was processed by the methods describe previously [55]. Briefly, 10 μg of solubilized proteins were separated on SDS-PAGE and electroblotted onto a polyvinylidene difluoride (PVDF) membrane (Bio-Rad Laboratories, Hercules, CA, USA). The membranes were blocked with 5% non-fat dry milk and probed sequentially with antibodies against Na^+^/K^+^ ATPase α-1 (1:500, Merck-Millipore 06-520, Darmstadt, Germany), and β-actin (1:5000, GeneTex GTX300041, Irvine, CA, USA). Following that, the PVDF sheets were incubated with HRP-conjugated secondary antibody at a dilution of 1:5000 for 1 h at room temperature. The immunoreaction was visualized with ECL solution (5 min) followed by 2 min of film exposure. The results of immunoblots were quantified using the computer-assisted software (Science Lab 2003, Fuji Film, Tokyo, Japan). All densitometric readings were normalized against β-actin and were presented as mean ± standard deviation (SD).

### Hippocampal bio-energetic mapping with [^14^C]-2-deoxyglucose (2-DG)

To evaluate the hippocampal bio-energetic activity, rats from each experimental groups were intraperitoneally injected with the activity marker 2-DG (100 μCi/kg; specific activity = 390 mCi/mmol.; VWR, Radnor, PA, USA) after ten days of GHB exposure. After 45 min. in their home cage, animals were deeply anesthetized and perfused transcardially as described above. Following perfusion, the brains containing the hippocampus were removed and cut into 30-μm-thick sections with a cryostat. The collected sections were batch-processed by affixing them to Bristol board, and apposing them to high-resolution X-ray film (Structurix, Agfa, Belgium) with [^14^C] microscales (Amersham, Piscataway, NJ, USA). Sections were exposed at -80 °C for ten days. After that, films were developed, fixed, and then digitized for the uptake of 2-DG followed the methods described previously^56^.

### Quantitative study and image analysis

The general approach for quantitative image analysis was similar to our previous studies^12^,^51^,^57^. A computer based image analysis system along with the Image-Pro Plus software (Media Cybernetics, Silver Spring, MD, USA) was used to quantify the staining intensity. A digital camera mounted on ZEISS microscope (Axioplane 2, Carl Zeiss MicroImaging GmbH, Hamburg, Germany) imaged sections and displayed them on a high-resolution monitor. The hippocampal neurons reacted for NMDAR1, p-CREB, and COX were densitometrically measured, and all readings were combined and averaged to obtain the total OD (TOD). Background staining (BOD) was measured on ventricular spaces adjoining the hippocampus. True OD was expressed by subtracting the BOD from TOD, so that each measurement was made in an unbiased way to correct for background. All images were captured on the same day by the same experimenter to maintain the uniform settings adjusted at the beginning of capturing. As the actual amount of reaction product deposited in a tissue section as a result of enzyme activity is influenced by a variety of factors, all parameters were carefully controlled following the recommended procedures for gray level adjustment, histogram stretch and minimal OD^58^.

### Statistical analysis

For TOF-SIMS analysis, spectral intensity detected from each section (with 10 sections per animal and nearly 80 cells per section) were normalized to ion intensity of paraformaldehyde (serve as base line = 100%) and expressed as percentage above the base line^55^. All of the normalized spectra collected from each animal were then averaged to obtain the representative data for that animal. The representative data acquired from animals belonging to the same experimental group (n = 6) were further averaged to yield the mean value for that corresponding group^55^. Comparisons among mean values obtained from different experimental groups and other data acquired from spectrometric, biochemical and neurochemical methods were subjected to Kolmogorov-Smirnov test for analyzing the pattern of normality. Those qualified and the behavioral data were subsequently processed for repeated-measures ANOVA (date of escape latency vs. behavioral changes along training) followed by Bonferroni *post hoc* test. Statistical difference was considered substantial if *P* < 0.05.

## Additional Information

**How to cite this article**: Youn, S.-C. *et al.* Comprehensive Application of Time-of-flight Secondary Ion Mass Spectrometry (TOF-SIMS) for Ionic Imaging and Bio-energetic Analysis of Club Drug-induced Cognitive Deficiency. *Sci. Rep.* **5**, 18420; doi: 10.1038/srep18420 (2015).
